# Enhancing RNAi Efficiency to Decipher the Functional Response of Potential Genes in *Bemisia tabaci* AsiaII-1 (Gennadius) Through dsRNA Feeding Assays

**DOI:** 10.3389/fphys.2020.00123

**Published:** 2020-03-02

**Authors:** Ramandeep Kaur, Mridula Gupta, Satnam Singh, Neelam Joshi, Abhishek Sharma

**Affiliations:** ^1^Department of Microbiology, Punjab Agricultural University, Ludhiana, India; ^2^Regional Research Station, Punjab Agricultural University, Faridkot, India; ^3^Department of Entomology, Punjab Agricultural University, Ludhiana, India; ^4^Department of Vegetable Sciences, Punjab Agricultural University, Ludhiana, India

**Keywords:** nanoparticles, gut nucleases, knockdown, *Bemisia tabaci*, virus transmission, mortality, osmoregulation

## Abstract

Whitefly *Bemisia tabaci* is a global invasive pest that causes substantial losses to agricultural crops worldwide either by direct feeding or vectoring numerous plant viruses. Management with insecticides remains a big challenge due to its rapid resistance development potential as well as the impact of these chemicals on non-target organisms. Thus, in search of alternate and novel pest management strategies RNA interference (RNAi) has come up as potential future tool in this direction. The present study targets nine potential genes (Aquaporin (*AQP*), Calcitonin (*CAL*), CyclophilinB (*CYCP*), Knottin-1 (*k-1*), Heat shock proteins (*Hsp20*, *Hsp40* and *Hsp70*), SWItch/Sucrose Non-fermentable (*SNF7*) and inhibitor of apoptosis (*IAP*) of whitefly that have been implicated to play a role in various vital physiological functions and virus transmission. The RNAi mediated knockdown efficiency of these genes has been improved through the conjugation of respective target gene dsRNA with CQD (carbon quantum dots) nanoparticles or simultaneous knockdown of dsRNA specific gut nucleases. The studies revealed that feeding of dsRNA (40 μg/ml sucrose diet) of the target gene(s) either conjugated with CQD or along with dsRNA against *dsRNase* (dsdsRNase) (40 μg/ml sucrose diet) enhanced the RNAi efficiency by 24–89% compared to whiteflies fed with naked dsRNA of the same target gene. The studies provide insights about the functional role of various genes in whitefly, which can possibly be exploited for the management of this pest in the future.

## Introduction

*Bemisia tabaci* (Gennadius) (Hemiptera: Aleyrodidae) is a cosmopolitan pest capable of feeding on hundreds of plant species and transmits several major plant viruses (Brown, 2000; Navas-Castillo et al., 2011; Rosen et al., 2015). *B. tabaci* has gained the status of key pest of economic importance due to wider host range, dispersal behavior, fecundity, competency in virus transmission, and insecticide resistance (Ahmad et al., 2002; Boykin et al., 2007). During 2015–2016, the massive infestation of whitefly in north India resulted in huge loss to cotton crop. There was complete failure of all the management strategies including the mainstay chemical insecticides to control this pest due to resistance development or substandard pesticides. Biotechnological tools in recent times have been successful in providing pest management solutions such transgenic Bt-cotton expressing Cry proteins against bollworms. Such biotechnological tool RNA interference (RNAi) has emerged as a revolutionary technology for exploring novel targets in insects from that can be potentially exploited for their management. The major advantage of this technology over present transgenic is that the chances of insects developing resistance to dsRNA are very rare. RNAi is an antiviral mechanism that leads to activation of defense response, which recognize the corresponding homologous, double-stranded RNA (dsRNA) and targets it for enzymatic degradation (Mello and Conte, 2004). The mechanism involves the introduction of exogenous or experimental dsRNA in the host cell (Winston et al., 2002) followed by the cleavage of long dsRNA into 21–25 bp siRNA (Bernstein et al., 2001) by *Dicer* enzyme which is *RNaseIII* type nuclease. The duplex complex of siRNA is recruited by a multi-protein complex called RNA-induced silencing complex (RISC) where passenger strand tagged for degradation and guide strand of siRNA along with argonaute proteins bind to complementary mRNA in a sequence specific manner and leads to its degradation (Scott et al., 2013). This results in the modulation of target protein by depletion of target mRNA either by transcriptional or at a post-transcriptional level. Thus, RNAi has the potential to identify novel genes whose knockdown is detrimental to the target insect. RNAi has been well established in many insect species such as pea aphid (*Acyrthosiphon pisum*) (Jaubert-Possamai et al., 2007), leafhopper (*Nilaparvata lugens*) (Zha et al., 2011), flour beetle (*Tribolium castaneum*) (Whyard et al., 2009), kissing bug (*Rhodnius prolixus*) (Taracena et al., 2015), African malaria mosquito (*Anopheles gambiae*) (X Zhang et al., 2010), Cotton leafhopper (*Amrasca biguttula biguttula*) (Singh et al., 2018), cotton mealybug (*Phenacoccus solenopsis*) (Singh et al., 2019), Fruitfly (*Drosophila melanogaster*) (Schwedes and Carney, 2012). Recently the corn plants expressing dsRNA against reproductive genes of *Diabrotica virgifera virgifera* has demonstrated the potential of this technology through RNAi based transgenic plants (Niu et al., 2017). Alternatively, dsRNA-based sprays under curtailed conditions have also been proven to be effective against different insects (Palli, 2014; Cagliari et al., 2019).

RNAi has been well established in whitefly either through injection (Ghanim et al., 2007; Luan et al., 2011), oral route (Vyas et al., 2017) or by expressing their homologous double stranded RNAs in plants (Malik et al., 2016). Delivery of siRNA/dsRNA against genes like, α*-tubulin, V-ATPase, Cyp315a1, EcR, E75, Cyp18a, jhe, P450 CYP6CM1,Hsp70, RPL9, Glutathione S-transferase GS*T gene and *ADP/ATP translocase* has been studied in case of *B. tabaci* (Luan et al., 2013; Upadhyay et al., 2013; Li J. et al., 2015). The dsRNA expressed in entomopathogenic fungi, *Isaria fumosorosea* has also been reported to induce silencing of target genes in the insect host (Chen et al., 2015). In all these studies the extent of knockdown achieved across targeted genes ranged between 60 and 90% compared to controls. This is because the success and efficacy of RNAi depends upon several factors which include presence of core RNAi machinery, dose of dsRNA, the mode of RNAi delivery (Roignant et al., 2003), and the genes being targeted (Kitzmann et al., 2013). RNAi response is highly variable among insect species with lepidopteran being toward the lowest side and coleopterans showing highest efficiency (Shukla et al., 2016; Singh et al., 2017). The variability may be due to several factors, but majorly an impaired or slow cellular uptake of dsRNA in the gut and degradation of dsRNA in the insect body lead to deficient RNAi response. In hemipteran insects the dsRNA specific gut nucleases play a key role in the degradation of ingested dsRNA before it reaches the target cell (Arimatsu et al., 2007; Luo et al., 2017).

In order to develop an efficient RNAi-based method to control whiteflies it is important to protect the dsRNA from proteolytic degradation in the insect gut. Conjugation of target gene dsRNA with nanoparticles can protect the dsRNA against nucleolytic degradation and prolong the stability of dsRNA in the gut long enough to allow enough cellular uptake by the midgut cells (Xiang et al., 2012; Li M. et al., 2015; Dhandapani et al., 2019). Various studies have evidenced the potential usage of nanoparticles to improve RNAi in insects through feeding which are otherwise refractory to RNAi. Targeting dsRNA incorporated with guanylated nanoparticles against *chitinase synthase B* resulted in improved efficiency of RNAi in *Spodoptera exigua* (Christiaens et al., 2018) and *S. frugiperda* (Parsons et al., 2018) chitosan coated dsRNA resulted in 40% downregulation of axon guidance gene *semaphorin-1a* in *Aedes aegypti* (Zhang et al., 2015). The other alternative to nanoparticles is the simultaneous or consecutive silencing of *dsRNases* specific genes along with the target gene to restrict synthesis of dsRNses in the gut, which will in turn help to improve the RNAi efficiency (Spit et al., 2017; Guan et al., 2018). Thus, the current studies explore both the strategies i.e., conjugation of dsRNA with CQD nanoparticles and knockdown of dsRNA specific gut nucleases (dsRNases) for enhancement of RNAi of genes associated with various physiological functions and validated the same through various assays.

## Materials and Methods

### Whitefly Culture Maintenance and Collection

Whitefly haplotype Asia-II-1 was reared on cotton (*Gossypium hirsutum*) plants and maintained in an insect-proof glass house at 26°C with a relative humidity of 60 ± 70% and 14:10 h light and dark period.

### Extraction of Total RNA and cDNA Synthesis

Total RNA from whitefly was extracted using Trizol reagent (Sigma Aldrich) as per manufacturer’s protocol. For this 15–20 whiteflies were homogenized in 1 ml of trizol reagent. The DNA traces were removed from the total RNA by treating it with 1U of *DNase I* (Fermentas, ThermoFisher Scientific) for 30 min at 37°C followed by enzyme inactivation using EDTA and incubation at 70°C for 10 min. A total of 500 ng RNA was used for cDNA synthesis with RevertAid First Strand cDNA synthesis kit (Thermo Fisher Scientific) as per manufacturer’s instructions. The cDNA was stored at –20°C until further use.

### *In vitro* dsRNA Synthesis

The target gene sequences were pulled out from whitefly genome database^1^. Selection of genes was done on the basis of their functional role i.e., osmoregulation (*Aquaporin* (*AQP*) (Benoit et al., 2014), *Calcitonin (CAL)* (Furuya et al., 2000; Cohen, 2012; Zandawala, 2012), virus transmission (*Cyclophilin* B (*CYCP*) (Kanakala and Ghanim, 2016), *Knottin* (*k-1*) (Hariton Shalev et al., 2016), *Heat shock proteins* i.e., *Hsp20*, *Hsp40* and *Hsp70* (Gotz et al., 2012; Gorovits and Czosnek, 2017) and other genes associated with vital processes i.e., *SNF7* (*SWItch/Sucrose Non-Fermentable*) and *IAP* (*inhibitor of apoptosis*) (Blitvich et al., 2002). The expression of these genes was validated using gene specific primers (designed using Primer3 software) (Table 1) in qRT-PCR performed using LightCycler^®^ 96 System (Roche life sciences, Mannheim, German) with SYBR-Green detection (SYBR^®^ Premix Ex TaqTMII, Takara). The reaction was as follows: 30 s at 94°C followed by 40 cycles consisting of 10 s at 94°C, 30 s at 55°C, and 20 s at 72°C. The template for dsRNA (300–500 bp) against respective gene was amplified from cDNA using gene specific primers having T7 promoter sequence (5′-TAATACGACTCACTATAGGG-3′) at the 5′ and 3′ends of both reverse and forward primer. Amplicons were gel purified using Macherey-Nagel^TM^ NucleoSpin^TM^ Gel and PCR Clean-up Kit as per manufacturer’s protocol and used as template for *in vitro* transcription using TranscriptAid T7 High Yield Transcription Kit (Fermentas) as per kit manual. The dsRNA quality and quantity was determined by agarose gel electrophoresis and Nanodrop quantification.

**TABLE 1:** Primers for the target genes selected for RNAi in *B. tabaci.*

Gene	dsRNA primers	qPCR primers
Hsp20	5′TAATACGACTCACTATAGGGAGCGTCAAATTCCAGTCACC3′	5′GGAGAAAATGTTTCCAACCGTA 3′
	5′TAATACGACTCACTATAGGGAGCGTCAAATTCCAGTCACC3′	5′ CTCAGAGAGCACAGATAGCTAA3′
Hsp70	5′TAATACGACTCACTATAGGGCTCGAAACAAGCGAGGAGATT3 ′	AGTGCGGACGAACTAGCACT
	TAATACGACTCACTATAGGGCGATGGAGAGCAGCATCAATTA	GCAGCCAAATGATCAAGTCA
Cyclophilin	5′TAATACGACTCACTATAGGGCAAGAGAGACAATCCAG3′	GACGTAGGTCAAGATCCAGAGA
	5′TAATACGACTCACTATAGGGCAAGAGAGACAATCCAG3′	GAGGAAACTGCTCGTCCTTT
SNF7	5′TAATACGACTCACTATAGGGCGTTGAAGAGGAAGAAGC3′	GGCCATGACATTGATGAAGATG
	5′TAATACGACTCACTATAGGGCCACTGCTGCAATTCTT3′	ATTGGCAGTGCTGGTGAA
Calcitonin	TAATACGACTCACTATAGGGCTGGTCACACTCTACGCATTT	GCCGTGAGAGCTACGTTTAT
	TAATACGACTCACTATAGGGGCTGTTGTCACCTCTCCATT	ATGTGACCAAGGCAGAGATG
dsRNAse	TAATACGACTCACTATAGGGGAAAGGGTCAAGGAGAGAAA	CGAACAACCCCTTCGAAAAA
	TAATACGACTCACTATAGGGGCTATGTCCTGACACAAAGT	CCACTCGCATTTGAGAGGAA
IAP	TAATACGACTCACTATAGGGCATCACGTCTTCCCAATCTTTT	GCACATAACTTGATCACCTCGAC
	TAATACGACTCACTATAGGGACGAGGCGATGTTCCTTGTT	CTGACTTCCCCAACTACGCTAC
Knottin-1	TAATACGACTCACTATAGGACGACCAAAGCTTATC	GGCAATTGTCTTCCTGACG
	TAATACGACTCACTATAGGATCTGGTTGTGCAATG	TGTGCAATGTCCAGGAGTTC
Hsp40	TAATACGACTCACTATAGGGGCTCAAGCGTACGAAGTCCTATC	5′CTGTAGAAAGGATCCC3′
	TAATACGACTCACTATAGGGGTGGTGATGATGGTGGTTACT	5′AAGTTCTTTCGCGCTTGG3′
AQP	TAATACGACTCACTATAGGGTAGTGCTCGTAGGATGCATGTC	GGACCGACATCAAAGGATCTAT
	TAATACGACTCACTATAGGGGACGGACTGGACAACTAAAAGC	CAATGTTTGTCCCAAATTCCAC

### dsRNA Degradation Studies

To study the impact of whitefly gut nucleases specifically *dsRNases* on orally delivered dsRNA, about 14 mg of whiteflies (∼*N* = 300) were taken in 200 μl of PBS (Phosphate buffer saline) and macerated softly to extract the hemolymph and gut juices in the buffer. Total protein was extracted in 100 μl of PBS with the help of 900 μl of 10% TCA (Trichloroacetic acid), followed by centrifugation at 8000 rpm for 10 min. The pellet was washed with three parts of ethyl ether and one part of ethanol to remove unwanted fat molecules and finally dissolved in 200 μl of 0.1 N NaOH. The total proteins were quantified with Folin Phenol reagent method (Lowry et al., 1951). The total protein concentration was estimated from crude hemolymph using spectrophotometer (Eppendorf) which were further serially diluted to half till four concentrations using nuclease free water followed by incubation of the dsRNA (1 μg) at 37°C for 1, 3, and 5 h. The degradation was estimated on the basis of integrity of residual bands on gel electrophoresis.

### Oral Delivery of *in vitro* Synthesized dsRNA

Adult whiteflies were collected by directly tapping leaf over 50 ml falcon tube which was cut down at the bottom and covered with muslin cloth (Upadhyay et al., 2011). Adult whiteflies released in tube were subjected to 2–3 h starvation prior to feeding on artificial diet. The dsRNA (400 ng) against different target genes (*AQP, CAL, SNF7, IAP, Hsp20, Hsp40, Hsp70, k-1, and CYCP*) was incorporated in 20% sucrose diet and sandwiched between two sterile layers of Parafilm M on the top side of 50 ml falcon tube. Whiteflies (30–35) in three different biological replicates were given the feeding access to mixture of dsRNA and sucrose diet for 48 h. Similar quantity of dsRNA against *green fluorescent protein gene* (dsGFP) was used as control.

Post 48 h of feeding accesses to dsRNA-sucrose diet mixture, about 25 whiteflies were collected in Trizol (Sigma-Aldrich) from each biological replicate followed by RNA isolation and cDNA synthesis as described in earlier section. The cDNA was diluted 10 times before setting the reaction and each qPCR reaction consisted of 1 μl cDNA, 0.1 μL of each primer (10 μM), and 5 μl of SYBR^®^ Premix *Ex Taq*^TM^ II in a total volume of 10 μl. The PCR parameters consisted of one cycle at 95°C for 30 s, followed by 40 cycles of 95°C for 5 s and 60°C for 30 s. The reactions were carried out in a LightCycler^®^ 96 Real-Time PCR System (Roche Applied Science). The relative quantification of genes was done using △△Ct method followed by Students’ *t*-test for testing the level of significance (*p* = 0.05). The expression data was normalized with β-tubulin as housekeeping gene and compared with dsGFP fed whiteflies as control.

### Enhancing RNAi

#### Knockdown of Gut Nucleases and Conjugation of dsRNA With CQD Nanoparticles

Gut dsRNases are major bottleneck for efficient RNAi in hemipteran insects as these are considered to be responsible for degradation of orally ingested dsRNA (Arimatsu et al., 2007; Allen and Walker, 2012; Liu et al., 2012). Protection against these nucleases in insect gut can be achieved either by known down of the gene(s) encoding for dsRNases or conjugation dsRNA with some nanoparticles. In the first approach dsRNA against dsRNases (dsdsRNases) was synthesized as per earlier described methodologies and fed (400 ng/μl sucrose diet) to the whitefly adults along with the target gene in three biological replicates. The second approach involved the preparation dsRNA-CQD (carbon quantum dots) nano-conjugate with target gene. For preparation of CQD, 9 ml of polyethylene glycol (M. W. 200; PEG-200) was mixed with 3 ml of water followed by addition of 100 mg of polyethylenimine (PEI) in 2 ml ddH_2_O. The mixture was heated in a microwave for nearly 3 min at 800 W till it turns light golden yellow. The cooled down PEG-PEI solution (100 μl) was mixed with 40 μg of target gene dsRNA in 100 μl of sodium sulfate solution followed by 4°C overnight incubation. Next day, the tubes were centrifuged at 12,000 rpm for 10 min and pellets were dissolved in 40 μl dd water. To confirm the conjugation of dsRNA with CQD, serial dilutions (1: 100; 1:200; 1:500 and 1:800) of this final CQD preparation were conjugated with the fixed amount of dsRNA (40 μg) and subjected to comparative gel retardation assay. It was anticipated that at a certain concentration CQD won’t be sufficient to retain/conjugate the dsRNA. Thus this dsRNA will appear down the agarose gel as visible in 1:500 and 1:800 dilution of CQD compared to the higher concentrations (1:100 and 1:200), which retained the dsRNA in the well. Thus it was confirmed that the CQD formed a conjugate with the dsRNA.

#### Conjugation of dsdsRNases and Target Genes With CQD Nanoparticles

The role of dsRNases and efficacy of the CQD particles was further confirmed by conjugating the dsRNA against dsRNase gene and feeding it along with target genes (*Hsp 40* and *IAP*) CQD-dsRNA conjugate. The CQD nanoparticles were prepared as described earlier and conjugated with 40 μg of dsdsRNase each separately with equal amount of dsHsp40 and dsIAP. The similar concentration of dsRNA against *GFP* was used to prepare ds*GFP*-CQD conjugate, which served as control. The conjugation of dsRNA and CQD prepared in each experiment was confirmed through Gel Retardation Assay (Das et al., 2015). Adult whiteflies (25–30) in four biological replicates were allowed to feed on different dsRNA-CQD conjugates incorporated in 20% sucrose diet. The total RNA from whiteflies in each treatment was isolated 48 h post feeding and reverse transcribed as per earlier described methodology. The relative expression of all the targeted gene (*Hsp40*, *IAP*, dsRNase) in each treatment was quantified by qRT-PCR with respective gene specific primers using 2^–ΔΔCT^ method after normalization with ß-*tubulin* as housekeeping gene (Kaur et al., 2019).

#### Validating the Impact of dsRNA Mediated Knockdown of *dsRNase* Gene on dsRNA Degradation in Hemolymph and Gut Juices

The gut nucleases such as dsRNases are known to hamper the RNAi efficiency by degrading the dsRNA in the insect gut. This was reconfirmed by the knockdown of *dsRNase* gene in whiteflies followed by extraction of haemolymph and gut juices and its consequent impact on dsRNA incubated with this extract. The whiteflies were allowed to feed on dsRNA (400 ng/μl of sucrose diet) against *dsRNase* gene synthesized as per earlier described protocols. Post 48 h of feeding accesses, the whiteflies were slightly macerated and processed for haemolymph and gut juice extraction as described in dsRNA degradation studies. The integrity of the dsRNA was assessed as per earlier described methodology.

### Estimation of Water Loss

The water loss after feeding of ds*AQP* and ds*CAL* was qualitatively estimated on water sensitive paper (Teejet Technologies, United States) disk (5 cm diameter), which turns blue on contact with any type of aqueous solution. The fluid excreted by the whitefly when in contact with this paper is estimated by the number of blue dots appearing on it. Whiteflies were released on the water sensitive paper 48 h post feeding of dsRNA for evaluating the silencing effect of these genes on fluid excretion. The qualitative assessment of the fluid excretion was done by the comparative evaluation of number of blue dots appearing on water sensitive paper in treatment and control. The quantitative estimation of fluid excretion was done using water loss monitoring system consisting of RM-8 Flow Multiplexer, RH-300 Relative Humidity analyzer, SS-4 gas analyzer sub-sampler pump and mass flow meter, RC-chambers and UI-3 A/D interface (Sable Systems International, United States). The control and target gene dsRNA (ds*AQP* and ds*CAL* (400 ng/μl sucrose diet) were fed to whiteflies in three biological replicates for 48 h followed by releasing counted number of insects in pre-conditioned (with N2 gas) RC chamber for 1 h. The amount of fluid excretion was sensed by the equipment and quantified use ExpeData software (Sable Systems International, United States).

### dsRNA Dose Mortality Relationship Calculated Using Probit Analysis

The dose-mortality relationship was calculated using serial concentrations of dsRNA against *SNF7* (ds*SNF7*) and *IAP* (ds*IAP*) genes. Different dsRNA doses against these genes, i.e., 100, 200, 400, and 800 ng/μl of sucrose diet along with ds*GFP* control were fed to adult whiteflies (∼100 in number) in three different replicates. The number of dead and live whiteflies were recorded 48 h post feeding and the data were subjected to Probit analysis (Higuchi et al., 1993) in POLO Plus software to calculate the LD_50_ values.

### Impact of Knockdown of *Hsp20*, *Hsp40*, *Hsp70*, *k-1*, and *CYCP* on Titer and Transmission of Cotton Leaf Curl Virus (CLCuV)

The impact of knockdown of *Hsp20*, *Hsp40*, *Hsp70*, *k-1*, and *CYCP* genes was evaluated through virus titer in whitefly and its transmission efficiency in cotton plants. The Cotton leaf curl Rajasthan virus (CLCuRv) (AB-2; KM098115.1) one of the most prevalent virus species (Geminiviridae: Begomovirus) in the region was maintained on susceptible cotton cv. RST9 at Regional Research Station, Faridkot and used as a source of inoculum in the study. The experiment was conducted in two separate batches. The first batch of viruliferous whiteflies (20–30) were allowed to feed for 48 h separately on dsRNA (400 ng/μl of diet) against different genes incorporated in sucrose diet as per earlier described methodology. The viral load in *B*. *tabaci* was quantified using qRTPCR with virus specific primers qCLCUV_F1 (5′CGTCGACCTGTTGATAAACCTC3′) and qCLCUV_R1 (5′GCATATTGACCACCGGTAACAG3′) with two technical replicates for each of the four biological replicates. After 48 h post feeding total RNA from each treatment was isolated and reverse transcribed into cDNA, which was used to quantify the transcripts using ΔCT method as per as per earlier described methodology. The whitefly β*-tubulin* gene (Gene ID: Bta0035 from whitefly genome with primers β-tublin-F: 5′- CACTGGTACGTAGGAGAAGGTA -3′ and β-tubulin-R: 5′-ACTGAGTCCATGCCAACTTC -3) was used as a reference gene for normalization of expression data (Kaur et al., 2019). To determine the implication of each gene in the transmission of CLCuV, the second batch of viruliferous *B. tabaci* (10 whiteflies/plant) were transferred on virus free cotton plants (three- four leaf stage) maintained in insect proof cages after feeding access of 48 h to each target gene dsRNA (400 ng/μl of diet) and sucrose diet mixture. Whiteflies allowed to feed only on 20% sucrose diet served as a control for this experiment. The experiment was conducted with four individual plants representing each replicate. Plants were continuously monitored for appearance of disease symptoms after 5 days of inoculation access period. After the appearance of early symptoms (formation of green islands) the top most young leaf was harvested in liquid nitrogen wrapped in aluminum foil and grounded to fine powder using a pestle and mortar and processed for total RNA isolation using NucleoSpin^®^ RNA Plant kit (Ref#740949.50) as per user guidelines. After DNase treatment for half an hour at 37°C and inactivation of enzyme at 70°C degrees. RNA purity and yield was analyzed using a spectrophotometer and 500 ng of total RNA was used for cDNA synthesis using PrimeScript^TM^ 1st strand cDNA Synthesis Kit (Cat no. 6110A). For quantification of the expression level of CLCuV, amplification was performed using ten-fold diluted cDNA as mentioned above using coat protein specific primers. The expression was normalized using RNA helicase gene (Table 1).

### Statistical Analysis

The relative expression was calculated using 2^–ΔΔCT^ method (Livak and Schmittgen, 2001) using suitable housekeeping gene for normalization. The comparative expression of target genes and GFP control was compared using Student *t*-test at *p* < 0.05. The expression data was presented means ±SEM.

## Results and Discussion

### dsRNA Degradation Studies and Enhancing RNAi Efficiency

**FIGURE 1:**
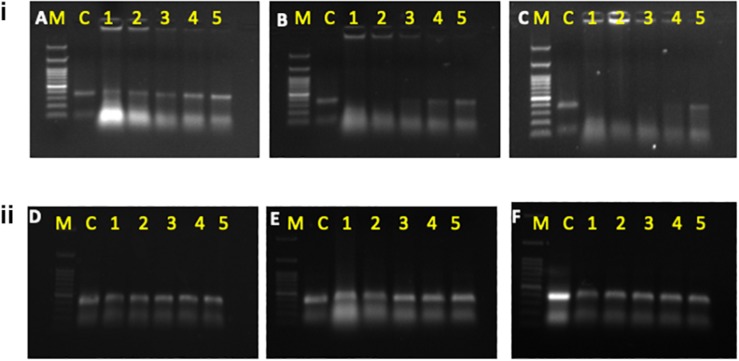
**(i)** Degradation of dsRNAat different concentrations of hemolymph at different time intervals i.e., 1 h **(A)**, 3 h **(B)**, and 5 h **(C)**. **(ii)** Degradation of dsRNA at different concentrations of hemolymph isolated from *dsRNases* knockdown whitefly insects at different time intervals i.e., l h **(D)**, 3 h **(E)**, and 5 h **(F)** M: 100 bp ladder, C, control dsRNA; 1, crude hemolymph (CH); 2, 1:1 dilution of CH; 3, 1:25 of CH; 4, 1:50 of CH; 5, l:100 of CH.

**FIGURE 2:**
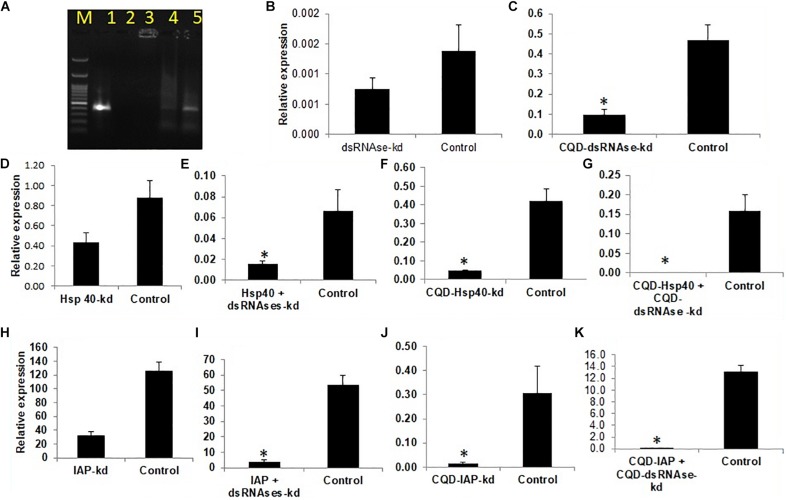
**(A)** Affinity of CQD nanoparticles at different dilutions with fived concentration of dsRNA.M -100 bp ladder 1- Control, 2-1:100,3-1:200,4- 1:500,5- 1:800, **(B)** *dsRNAses* knockdown- 45% **(C)** *CQD-dsRNAses-*79.08% **(D)** *Hsp40* knockdown 61.7% **(E)** *Hsp40* + *dsRNAses* knockdown -77.1% **(F)** CQD- *Hsp40* knockdown 88.9% **(G)** CQD- *Hsp40* + CQD-dsRNAse knockdown 99.4% **(H)** *IAP* knockdown 74.3*%* **(I)** *IAP* + *dsRNAses* knockdown- 92.54% **(J)** *CQD-IAP* knockdown 95.2% **(K)** CQD-*IAP* + CQD-dsRNAse knockdown 99.6%. The error bar represents the standard deviation (*n* = 3) and * represents significant differences in mRNA transcripts compared to the control (*P* ≤ 0.05, Student’s *t*-test).

The whitefly crude hemolymph comprised of 0.3 μg/ml of total protein which was further diluted to make 0.15 μg/ml, 0.07 μg/ml and 0.035 μg/ml concentration. Incubation of dsRNA with hemolymph and gut juices resulted in its degradation as indicated by the gel retardation assay (Figures 1A–C, The level of degradation was directly dependent on the concentration of hemolymph and gut juices as well as the period of incubation. The maximum degradation was observed in crude mixture (0.3 μg/ml of total protein), however, retention of integrity of dsRNA in higher dilutions signified the role of dsRNA specific nucleases in RNAi. The time of exposure was also a critical factor and directly proportional to dsRNA degradation. Prolonged period (5 h) of incubation resulted in more exposure of dsRNA to dsRNases consequently resulting in its degradation. When hemolymph was isolated from dsRNases knockdown whitefly, the incubated dsRNA didn’t showed notable degradation of dsRNA till 5 h (Figures 1D–F), which signifies the role of nucleases (dsRNases) in the hindrance of RNAi in whitefly. Various earlier reports also suggests the degradation of dsRNA in the insect belonging to lepidoptera, hemiptera, coleoptera and orthoptera orders due the presence of dsRNA specific nucleases in the gut or hemolymph (Singh et al., 2017; Peng et al., 2018; Prentice et al., 2019; Singh et al., 2019; Song et al., 2019). The conjugation of dsRNA with CQD was confirmed through gel retardation assay, which clearly indicated that an optimum concentration of CQD was required to conjugate the dsRNA (Figure 2A). The higher dilutions (1:500 and 1:800) of original CQD concentration were not sufficient to retain the dsRNA in the well of agarose gel and thus it was visualized down the gel equivalent to the naked dsRNA. Thus confirming that optimum concentration of CQD successfully conjugated with dsRNA and retained it in the agarose well. It is presumed that conjugation of dsRNA with CQD and its incubation in hemolymph and gut juices successfully protected the dsRNA from nucleolytic degradation. The gel retardation assay clearly indicated the retention of dsRNA-CQD conjugate in the agarose gel well (Figure 2A). The gut nucleases in hemipteran insects are reported as major bottleneck in efficient RNAi, as these enzymes hamper the concentration of dsRNA reaching the cell (Huvenne and Smagghe, 2010; Joga et al., 2016). Besides hemipteran, the degradation of dsRNA by dsRNases have also been reported from other insect orders (Almeida Garcia et al., 2017; Singh et al., 2017; Prentice et al., 2019; Song et al., 2019). Our studies successfully demonstrated the degradation of dsRNA in hemolymph and gut juices of whitefly. Further the conjugation of dsRNA with CQD was helpful in protecting dsRNA against the degradation by these nucleases. Protecting the dsRNA against dsRNases gene with CQDs helped in increasing the knockdown efficiency of this gene from 45.0 to 79.08% (Figures 2C,D). Furthermore, conjugation of dsRNA against dsRNase gene with CQD and administering it to whiteflies along with dsRNA against Hsp40 and IAP resulted in higher knockdown efficiency i.e., 99.4% and 99.6% of both the genes, respectively, compared to control (Figures 2G,K). Although RNAi is a well implemented strategy in whiteflies for gene knockdown (Vyas et al., 2017) but due to some major factors such as degradation of dsRNA due to some nucleases poses constraint to sensitivity of the process in vivo (Luo et al., 2017). The uptake of dsRNA in hemiperetan insect’s cells needs validation about the systemic spread of RNAi, which may occur either through SID-1 receptors or clathrin mediated endocytosis. Clathrin-endocytosis mediated uptake of dsRNA toward cellular membrane is a very slow cellular process which can enhanced by nanoparticle mediated delivery of dsRNA (Joga et al., 2016). Nanoparticles act as molecular carriers for dsRNA delivery thereby increasing the persistence of dsRNA and cellular uptake (Das et al., 2015). Several nanoparticles such as liposomes, guanylated polymers, quantum dots, branched amphiphilic peptide capsules (BAPCs), core shell nanoparticles have been used in various insects to enhance the RNAi effect (Avila et al., 2018; Zhang et al., 2015; Christiaens et al., 2018). The association of between nanoparticles and target dsRNA is mediated by electrostatic interaction which involves binding of amino group of nanoparticle with the phosphate group of dsRNA (Kunte et al., 2020). The conjugation between dsRNA and CQD particles may be due to polyethyleneimine (PEI) which is a cationic polymer and possess binding affinity to the nucleic acid to form a particulate complex which results in a decrease in the size of target dsRNA resulting in faster transfection efficiency and improved stability (Tros de Ilarduya et al., 2010). Another plausible explanation may be the ability of CQD particles to induce buffering causing osmotic rupture of endosomes and leading to internalization of dsRNA into the cytoplasm of the gut cells (Ma, 2014). CQD nanoparticles have been used for efficient delivery of dsRNA targeted against glyceraldehyde-3-phosphate dehydrogenase gene (G3PDH) to induce gene knockdown and systemic delivery of dsRNA against rice striped stem borer (Chilo suppressalis) (Wang et al., 2019). These nanoparticles have also been demonstrated as efficient carriers for dsRNA with high retention capability and delivery leading to gene silencing and mortality in A. aegypti (Das et al., 2015). Our studies have systematically demonstrated the degradation of dsRNA in whitefly body fluids due to the presence of dsRNA specific nucleases, which have been earlier characterized from B. tabaci (Luo et al., 2017). Both conjugation of dsRNA with CQDs and knockdown of dsRNases proved efficient ways for improving RNAi efficiency of any target gene. The conjugation of dsRNA with nanoparticles like CQD will boost up gene function studies by enhancing the RNAi efficiency, which in turn will be helpful to visualize the exact impact of particular gene knockdown on whitefly. On the other hand, the enhancement of RNAi through knockdown of gut nucleases can be used in future pest management strategies based on dsRNA sprays and transgenic plants. The dsRNA against dsRNases gene can be sprayed or expressed in transgenic plant along with target gene dsRNA to have an efficient whitefly management strategy.

### Knockdown of Osmoregulatory Genes

**FIGURE 3:**
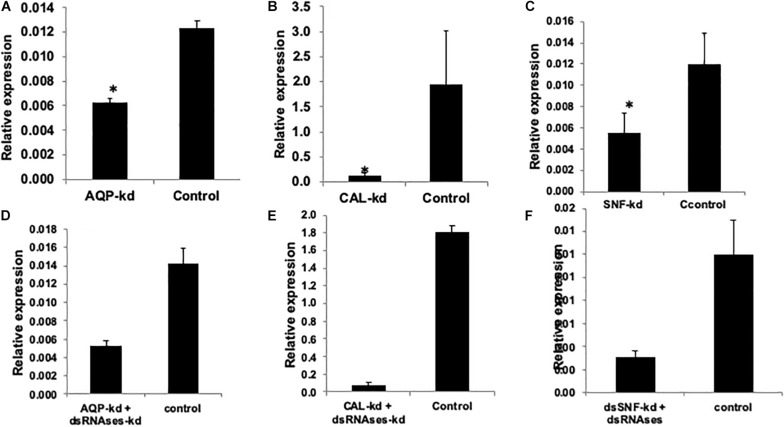
Relative expression of *AQP, CAL, SNF*, and *IAP* compared to GFP control when normalized with *tubutin.* **(A)** *AQP* knockdown (36.9%), **(B)** *CAL* knockdown (93.27%), **(C)** *SNF* 53.2%, **(D)** *AQP* + *dsRNAses* knockdown (62.7%), **(E)** *CAL* + *dsRNAses* knockdown (95.57%) **(F)** *SNF* + *dsRNAses* 74.16% The error bar represents the standard deviation (*n* = 3) and *represents significant differences in mRNA transcripts compared to the control (*P* ≤ 0.05, Student’s *t*-test).

**FIGURE 4:**
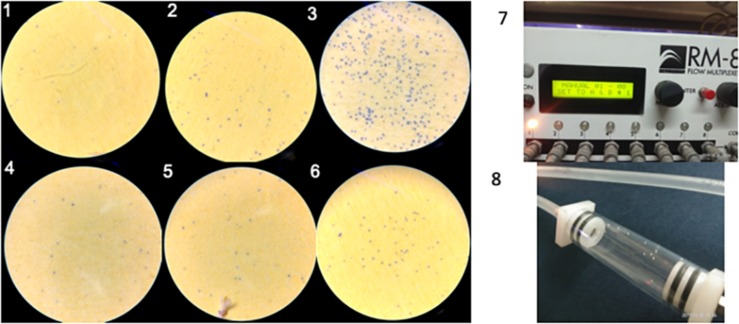
**(1–3)** Water spots calculated in *AQP* + *dsRNAses* knockdown ts 0.039 spots/mm^2^, *AQP* knockdown is 0.08 spots/mm^2^ as compared to *GFP* control is 0.25 spots/mm^2^, respectively. **(4–6)** water spots calculated in *CAL* + *dsRNAses* knockdown is 0.03 spots/mm^2^, *CAL* knockdown is 0.08 spots/mm^2^ as compared to *GFP* control is 0.16 spots/mm^2^, respectively. **(7)** Water vapor analyzer (RM-S flow multiplexer) to study water loss from the whitefly. **(8)** whitefly released in the channel of RM-8 flow multiplexer to estimate water loss.

Two genes *Aquaporion* (*AQP*) and *Calcitonin* (*CAL*) were targeted to functionally validate their role in osmoregulation in whitefly through their dsRNA mediated knockdown. Feeding of *dsAQP* and *dsCAL* led to 1.9 and 14.7 fold reduction mRNA level of *AQP* and *CAL* in whitefly, respectively (Figures 3A,B). Further enhancing RNAi by feeding ds*dsRNase* along with ds*AQP* and ds*CAL* in each treatment resulted in 2.9 and 22.5 fold reduction in mRNA levels of *AQP* and *CAL*, respectively (Figures 3D,E). Aquaporins are *trans*-membrane proteins which have a role in excretion of fluids and heat tolerance (Cohen, 2012; Benoit et al., 2014). On the other hand, Calcitonin is a neuropeptide which is responsible for the transportation of salt and water with the help of Malpighian tubules in the insects (Zandawala, 2012). The qualitative assessment of water loss in AQP and *CAL* silenced whiteflies was quantified on water sensitive paper, which revealed least fluid excretion (0.039 spots/mm^2^) in *AQP* + *dsRNAses* knockdown followed by sole *AQP* knockdown (0.08 spots/mm^2^) compared to GFP control (0.25 spots/mm^2^) [Figures 4(1–3)]. Similarly, the blue spots calculated in *CAL* + *dsRNAses* knockdown were 0.03 spots/mm^2^, while 0.08 spots/mm^2^ were observed in *CAL* alone knockdown whiteflies were as compared to 0.16 spots/mm^2^ in *GFP* control [Figures 4(4–6)]. The quantitative fluid loss estimated through humidity analyzer revealed that the knockdown of ds*AQP* and ds*CAL* led to excretion of 123 and 86 nl/min per whitefly compared to 203.15 nl/min per whitefly in ds*GFP* control whiteflies [Figures 4(7–8)]. Earlier studies with knockdown of *AQP* in *Cimex lectularius* also observed in 50% reduction in water excretion (Tsujimoto et al., 2017). The knockdown of *AQP* and *CAL* in *Phenacoccus solenopsis* also witnessed reduction in fluid loss compared to *GFP* control (Singh et al., 2019). Since *Calcitonins* are responsible for diuresis in insects (Furuya et al., 2000), the RNAi mediated knockdown of *calcitonin receptor* in *Aedes aegypti* led to significant reduction in fluid excretion as compared to ds*GFP* control insects (Kwon et al., 2012) and enhanced mosquito desiccation resistance (Drake et al., 2015).

### Dose-Mortality Relationship Vis-a-Vis Knockdown of *SNF7* and *IAP*

Feeding of 400 ng/μl of ds*SNF7* and ds*SNF7* + ds*dsRNase* caused 53.2 and 74.1% decrease in expression of *SNF7* gene compared to *GFP* control (Figures 3C,F). The knockdown of *IAP* further demonstrated significant suppression in mRNA levels of this gene with ds*IAP* + ds*dsRNaseII* (92.54%) and ds*IAP*-CQD nanoconjugate (95.2%) when compared to naked ds*IAP* (74.3%) and GFP control (Figures 2H–J). The lethal effects of ds*IAP* and ds*SNF* estimated through dose-mortality relationship suggested that 100 ng/μl dose of any of dsRNA against these gene could not cause any mortality of whitefly. The LD_50_ of ds*IAP* and ds*SNF* was 1023.9 and 1062.1 ng, respectively, while 4713.6 ng of IAP and 10834 ng of ds*SNF* was required for causing mortality in 90% of the treated whitefly population (LD_90_). *SNF7* is responsible for relieving major obstacle during transcription occurring due to nucleosome genome packaging. The SWI-SNF complex triggers chromatin remodeling via conformational or positional changes of nucleosomes, thus providing the access of transcriptional machinery to target genes (van Attikum et al., 2004; Nguyen et al., 2017). Additionally, *SNF7* complexes have potential role in DNA double-strand breaks repair and nucleotide excisions (van Attikum et al., 2004). Earlier reports on bed bugs (Basnet and Kamble, 2018) and mealybug bug (Singh et al., 2019) suggested induction of mortality when injected with *SNF7* specific dsRNAs. However, the amount of dsRNA against a particular gene that may cause a certain level of mortality may strictly depend its vitality or role that gene plays in an organism. There was not much variation between LD_50_ dose of ds*IAP* and ds*SNF7* but the difference between LD_90_ doses of two genes was almost half for ds*IAP* compared to dsSNF. *IAP* is known to block apoptosis in mammalian cells and its functional analysis of this gene has been studied in many insect species (Q. Li et al., 2005; Singh et al., 2018, 2019). *IAP* proteins provide a fundamental restriction to apoptosis (Vandergaast et al., 2015), thus their knockdown in insects or insect cell lines has resulted in their mortality at variable doses (Rodrigues et al., 2017; Cao et al., 2018).

### Impact of Knockdown of *Hsp’s*, *k-1*, and *CYCP* Genes in Whitefly on Virus Transmission Efficiency in Cotton Plants

**FIGURE 5:**
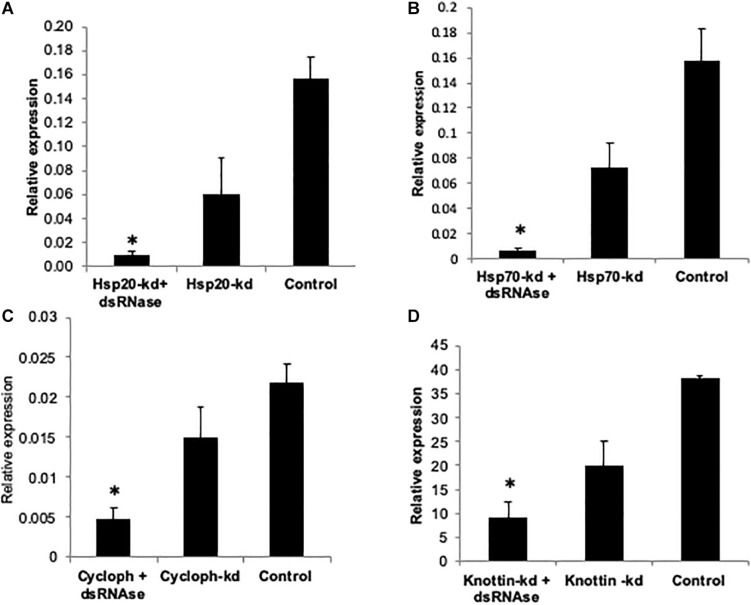
Relative expression of **(A)** *Hsp20* **(B)** *Hsp70* **(C)** *Cyclophitin* **(D)** *Knottin-1*, compared to GFP control when normalized with *tubulin.* The error bar represent the standard deviation (*n* = 3) and ^∗^represents significant differences in mRNA transcripts compared to the control (*P* ≤ 0.05, Student’s *t*-test).

Comparative knockdown effect of different genes associated with virus transmission was evaluated through feeding of respective naked dsRNA as well as its conjugation with Carbon Quantum Dots (CQDs) and simultaneous knockdown dsRNA specific (dsRNase) gut nuclease gene. As described earlier CQD-dsRNA conjugation and *dsRNase* gene knockdown was attempted with the aim to provide protection to dsRNA against nucleolytic degradation in the whitefly gut and its enhanced stability to allow sufficient cellular uptake. The qRT-PCR results clearly indicated that the feeding with target gene dsRNA (40 μg/ml sucrose diet) either conjugated with CQD or along with dsRNA against dsRNase (dsdsRNase) (40 μg/ml sucrose diet) enhanced the RNAi efficiency compared to whiteflies fed with only dsRNA (naked dsRNA against the same target gene) as well as control (dsRNA against *green fluorescent protein* gene- ds*GFP*). Feeding whitefly with ds*Hsp40*, ds*Hsp40* + ds*dsRNase* and ds*Hsp40*-CQD resulted in 61.7, 77.1, and 70.0% knockdown of mRNA levels compared to control whiteflies, respectively (Figures 2D–F). The results further suggested that among CQD conjugated dsRNA and parallel knockdown of dsRNAse, the RNAi efficiency of latter had a numerical edge over CQD-dsRNA conjugate in case *Hsp40* genes. Feeding whiteflies with ds*Hsp20* and ds*Hsp20* + ds*dsRNase* resulted in 61 and 92.2% reduction in mRNA levels when compared to *GFP* control (Figure 5A). The knockdown of *Hsp70* with naked dsRNA and ds*Hsp70* + dsRNAse caused 65.5 and 95.9% reduction in mRNA levels, which was significantly lower as compared to *GFP* control (Figure 5B). The coadministration of ds*Hsp70* along with dsdsRNaes resulted in significant reduction (88.3%) in the expression of *Hsp70* as compared to naked ds*Hsp70*. For the past few years RNA interference (RNAi) has emerged as a promising alternative technology to suppress crop pests and its application toward pest management is already close to a reality (Palli, 2014; Zotti and Smagghe, 2015). However, the success of RNAi depends upon the persistence of double-stranded RNA in insect hemolymph. Nonetheless several insect species mainly lepidopteran insects have been observed totally refractory to RNAi due to degradation of exogenous dsRNA (Garbutt and Reynolds, 2012; Shukla et al., 2016). Many studies suggest the role of gut nucleases specifically dsRNases in poor RNAi response in insects (Singh et al., 2019). The success rate of RNAi in earlier studies on *B. tabaci* leads to partial knockdown of the target genes thereby posing major constrained for RNAi in order to be used as a potent strategy for the management of whiteflies (Ghanim et al., 2007; Upadhyay et al., 2011; Raza et al., 2016). Several studies from the past evidenced that gut nucleases are the major factor limiting the efficacy of orally delivered dsRNA in insects (Arimatsu et al., 2007; Liu et al., 2012).

**FIGURE 6:**
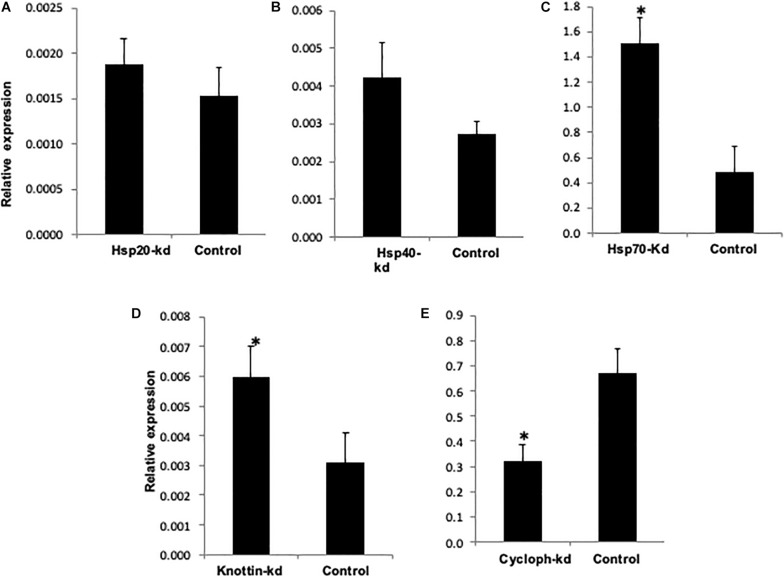
Relative amount of CLCuV (normalized to the host β- *tubutin* gene) after 48 h of feeding in **(A)** *Hsp20* knocked out whiteflies. **(B)** *Hsp40* knocked out whiteflies. **(C)** *Hsp70* knockout whiteflies. **(D)** *Knottin-1* knocked out whiteflies. **(E)** *Cychphilin* knocked out whiteflies. *represents significant differences in mRNA transcripts compared to the control (*P* ≤ 0.05, Student’s *t*-test).

**FIGURE 7:**
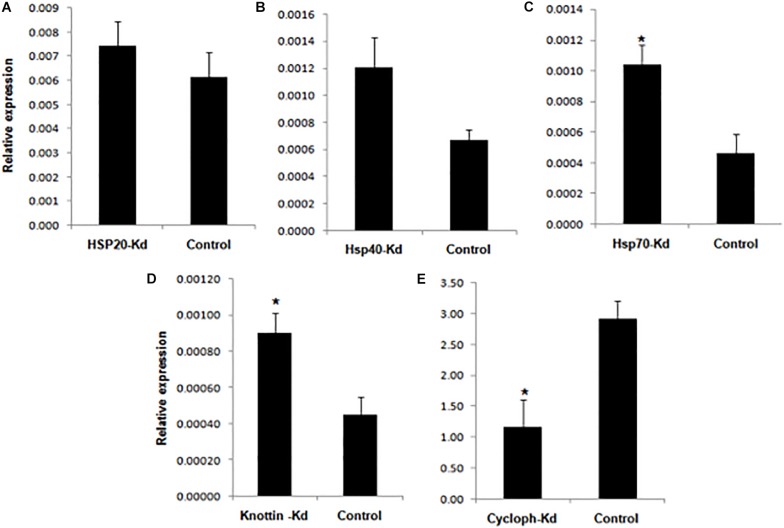
Relative amount of CLCuV (normalized to the cotton RNA helicase gene after 14 dpi with whiteflies fed with dsRNA + dsRNAase corresponding to **(A)** *Hsp20* **(B)** *Hsp4G* **(C)** *Hsp70* **(D)** *Knottin-1* **(E)** Cyclophilin. *represents significant differences in mRNA transcripts compared to the control (*P* ≤ 0.05, Student’s *t*-test).

The results showed that knockdown of dsRNAse caused significant reduction in transcripts of target genes compared to ds*GFP* control and naked dsRNA against the target genes. Earlier reports also suggest that in phloem feeding insects such as *B*. *tabaci* effectiveness and success of RNAi against specific genes can be improved by combination with RNAi against the dsRNase genes (Luo et al., 2017). Our results also indicate that dsRNase may be considered as a major barriers in limiting the efficacy of RNAi. Parallel to results, the depletion of nuclease leads to enhanced RNAi response in Colorado potato beetle *Leptinotarsa decemlineata* thereby increasing stability of oral delievered dsRNA (Spit et al., 2017). Similarly in migratory locust *Locusta migratoria*, RNAi mediated reduction of gene transcripts encoding dsRNAase significantly enhanced the RNAi process by oral delivery (Song et al., 2017, 2019). Altogether, RNAi-mediated knockdown of *dsRNase* genes emerged as a promising strategy for improving durability and efficacy of RNAi in various insect species (Scott et al., 2013; Wang et al., 2016). It has been also demonstrated that conjugation with different types of nanoparticles can help to protect the dsRNA in the insect gut thereby enhancing the RNAi efficiency of the target gene (Mysore et al., 2013; Zhang et al., 2015; Christiaens et al., 2018). However, the use of nanoparticles practically under field conditions have many regulatory issues and high input cost issues. Thus alternate strategy of dsRNases knockdown to enhance the RNAi efficiency of the target gene hold more potential and is feasible both in cases of dsRNA based sprays or plants expressing dsRNA. Keeping this in view the knockdown studies with other genes were done with parallel knockdown of *dsRNases* gene along with virus transmission associated genes. *Hsp’s* belong to multicomponent and multifunctional highly conserved molecular chaperone family involved in protein quality control. These prevent aggregation of damaged proteins, transportation, folding and unfolding, assembly and disassembly of multi-structured unit and assist degradation of misfolded proteins under stressed conditions (Lindquist, 1986; Parsell and Lindquist, 1993; Gregersen et al., 2001). This family of proteins is universally present in most of the organisms and has been classified and nomenclatured based on their molecular weight into *Hsp*100, *Hsp*90, *Hsp70, Hsp60, Hsp40, and* small Hsps (size >30 kDA), which is represented by the numeric prefix in the name of respective Hsp (Pelham, 1986). *Hsp70* comprises of ATP dependent most conserved family of proteins which are also known to play a major role in the biology of host and mediate circulative transmission of Begomoviruses (Mayer, 2005). Microarray based transcriptome analyses revealed that *Hsp70* was found to be overexpressed in response to virus acquisition and retention in whitefly (Gotz et al., 2012). The knockdown effect of these genes was also related to the virus titer in the knockdown host vector and its transmission efficiency to the cotton plants. Downregulation of Hsp70, *Hsp40* and *Hsp*20 resulted in significant increase i.e., 3.1, 1.5, and 1.2 fold in virus titer within the host vector compared to the control viruliferous whitefly (Figures 6A–C). Transmission efficiency of whitefly vector fed with dsRNA against *Hsp* genes assessed by the relative amount of virus titer in cotton leaves at 14 dpi showed significantly variation with the silencing of target genes. The amount of viral transcripts associated with test plants were measured 14 days post-inoculation. Whiteflies silenced with *Hsp70* showed significantly increased (2.2 fold) transmission efficiency when compared to control plants (Figure 7). Numerically higher transmission efficiency i.e., 1.2 and 1.7 fold was observed with the knockdown of *Hsp40* and *Hsp20* in whitefly, which otherwise was statistically at par with control (Figures 7A,B).

*Knottins* comprises structural family of proteins containing small disulfide rich proteins with a knotted appearance (Gelly et al., 2004). Whiteflies fed with ds*k-1* and combination of ds*k-1* + dsRNAse against *k-1* showed 47.6 and 76% reduction in expression of *k-1*, respectively, 48 h post-treatment compared to the *GFP* control (Figure 5D). The knockdown of *k-1* resulted in 1.9 fold increase in virus titer in the whitefly compared to the control whiteflies (Figure 6D). Further, the transmission efficiency of Cotton leaf curl disease (CLCuD) in cotton plants with these whiteflies was 2.0 fold higher as compared to ds*GFP* fed control whiteflies (Figure 7D). It may be possible that *Hsp’s* and *k-1* are involved in maintaining the amount of virus associated with whitefly vector to a level that may not be deleterious to host. Present study supports the fact that knockdown of *Hsp* and *knottin-1* might have led to the disruption of circulatory route of virion particle, which increased number of copies throughout the insect body and ultimately higher virus transmission efficiency in the inoculating test plant. In this study, inhibition of *Hsp70* expression resulted in a 55% increase in CLCuV transmission by *B. tabaci* thereby confirming previous results reporting inhibitory role of this gene against begomoviruses and act by minimizing the potential long-term harmful effects of the virus on the whitefly (Gotz et al., 2012). The role of *Hsp70* isoforms has also been reported in animal viruses like Dengue virus entry, replication and biogenesis in mosquito (Welsch et al., 2009). The knockdowns of *Hsp40* and *Hsp20* have also shown similar implication on virus titer in vector as well as its transmission in cotton host. Activity of both *Hsp70* and *Hsp40* is interdependent as *Hsp40* function as cochaperon acting as DnaJ dimer which stimulates the ATPase activity of *Hsp70* (Wickner et al., 1991; Jordan and McMacken, 1995). *Hsp20* belongs to the family of a 16-kDa protein also predicted to be involved in virus interaction (Ohnesorge and Bejarano, 2009). The downregulation of all these three genes result in increased virus titer in whitefly and increased virus transmission efficiency in the host plant. *Knottins* are found to be in abundance with diverse biological function. Silencing of *k-1* also led to significant increase in virus titer in vector *B. tabaci* which ultimately increased the percentage transmission by 50% in cotton plants at 14 dpi compared to control. These results are in accordance with previous report which used leaf mediated dip assay to target *k-1* gene in whiteflies and found that its downregulation led to increased amount of virus with in the whiteflies and tomato plants (Hariton Shalev et al., 2016). Hence, this study supports the earlier reports, which suggested that *Hsp and k-1* modulate virus titer in such a way which limits the virus titer to such a level so that it does not lead to any harmful effect inside whitefly body.

*Cyclophilins* belongs to a peptidyl–prolyl *cis*–*trans* isomerase (PPIases or Cyps) family of proteins which plays important role in protein refolding, maturation, cell signaling and gene transcription (Wang and Heitman, 2005; Hanes, 2015). The whiteflies fed with ds*CYCP* and ds + *dsRNAse* + *CYCP, s*howed ∼31.1% and 74.5% reduced mRNA levels of *CYCP* compared to the GFP control (Figure 5C). In comparison to *Hsp70, Hsp40, Hsp20*, and *k-1* lower number of viral transcripts were observed in *CYCP* knocked out whiteflies (Figure 6E). However, contrary to cotton plants infected with silenced *Hsp20, Hsp40, Hsp70, and k -1* whitefly which showed increased viral load, the plants infected with *CYCP* silenced whiteflies contained lower number of viral transcripts then the untreated plants (Figure 7E). The obtained results are consistent with previous reports in which knockdown of *cyclophilin* (CYCP) in *B. tabaci* showed lower efficiency to transmit tomato leaf curl virus (Kanakala and Ghanim, 2016; Kanakala et al., 2019).

## Data Availability Statement

The datasets generated for this study will not be made publicly available. There is no data set associated with this manuscript.

## Author Contributions

SS conceptualized and designed the experimental setup. RK and MG conducted the experiments, wrote the manuscript, and analyzed the data. NJ and AS contributed to data analysis and manuscript preparation.

## Conflict of Interest

The authors declare that the research was conducted in the absence of any commercial or financial relationships that could be construed as a potential conflict of interest.

## Footnotes

1 http://www.whiteflygenomics.org/cgi-bin/bta/index.cgi
